# 86. Access to Antimicrobial Susceptibility Testing for Novel Gram-Negative Antibiotics

**DOI:** 10.1093/ofid/ofab466.288

**Published:** 2021-12-04

**Authors:** Yuman Lee, Juliette Kim, Nicole Bradley

**Affiliations:** 1 St. John’s University College of Pharmacy and Health Sciences, Queens, New York; 2 Nassau University Medical Center, East Meadow, New York

## Abstract

**Background:**

Antimicrobial susceptibility testing (AST) is critical in identifying the optimal antimicrobial regimen for individual patients with serious gram-negative infections. Limitations to AST for newly developed antibiotics include the lack of commercially available AST methods, challenges of implementation due to regulations, and delays in obtaining results. The purpose of this study was to evaluate the access to AST for cefiderocol (FDC), imipenem-relebactam (IPR), meropenem-vaborbactam (MEV), and eravacycline (ERV) in hospitals across the U.S.

**Methods:**

An electronic survey was distributed to the American College of Clinical Pharmacist Infectious Diseases Practice and Research Network in May 2021. Hospital baseline demographics and current practices were collected.

**Results:**

Based on 50 responses, specimens were sent to in-house microbiology labs (37, 74%), core microbiology labs (8, 16%), and 3^rd^ party reference microbiology labs (5, 10%). AST for FDC was performed by 13 (35%) in-house labs, 4 (50%) core labs, and 1 (20%) reference lab. AST for IPR was performed by 11 (30%) in-house labs, 2 (25%) core labs, and 1 (20%) reference lab. AST for MEV was performed by 25 (68%) in-house labs, 3 (37.5%) core labs, and 1 (20%) reference lab. AST for ERV was performed by 1 (20%) in-house lab, 1 (20%) core lab, and 0 reference labs. 15 (30%) respondents were not able to get AST for any of the novel agents at their respective microbiology labs. Turn-around-times (TATs) for FDC, IPR, MEV, and ERV were ≥72 hours for 33 (66%), 35 (70%), 21 (42%), and 35 (70%) hospitals, respectively. When compared with 3^rd^ party reference labs, in-house labs with the ability to perform AST for these novel agents had significantly shorter TATs (p< 0.05). The average number of requests for AST for these novel agents was 20 times a year with an average of 113 minutes spent per patient on the coordination of AST.

**Conclusion:**

Access to AST for these novel agents varied across hospitals in the U.S. Nearly 1/3 of the respondents were not able to obtain AST for these agents at all. Long TATs exist and a great deal of time is spent per patient for coordinating AST for these novel agents. There is a crucial need for a multidisciplinary, collaborative approach to resolve the challenges in obtaining AST for newly developed antibiotics to provide patient care.

**Disclosures:**

**All Authors**: No reported disclosures

